# A lethal juvenile mouse model for the evaluation of antiviral reagents against coxsackievirus A4

**DOI:** 10.1016/j.isci.2025.113022

**Published:** 2025-06-30

**Authors:** Qin Su, Xiao Chen, Zhihui Zheng, Hailin Wei, Jiaxue Zhou, Libing Zhang, Ali Muhammad, Yin Wang, Xiang Chen, Pinghu Zhang

**Affiliations:** 1 Jiangsu Key Laboratory of Integrated Traditional Chinese and Western Medicine for Prevention and Treatment of Senile Diseases, Medical College, Yangzhou University, Yangzhou 225009, China; 2 Emergency Intensive Care Unit (EICU) and Department of Emergency Medicine, The Affiliated Hospital of Yangzhou University, Yangzhou University, Yangzhou, Jiangsu 225002, China; 3 National Human Diseases Animal Model Resource Center, NHC Key Laboratory of Human Disease Comparative Medicine, National Center of Technology Innovation for Animal Model, Institute of Laboratory Animal Sciences, Chinese Academy of Medical Science and Peking Union Medical School, Beijing 100021 China; 4 Yangzhou Center for Disease Control and Prevention, The Affiliated CDC of Yangzhou University, Yangzhou 225001, China; 5 Jiangsu Key Laboratory of Zoonosis, Jiangsu Co-Innovation Center for Prevention and Control of Important Animal Infectious Diseases and Zoonoses, Yangzhou University, Yangzhou, Jiangsu 225009, China

**Keywords:** pharmacology, microbiology, virology

## Abstract

Coxsackievirus A4 (CVA4) as a pathogen causing severe herpangina has become global outbreak epidemics during the past 5 years. However, there is currently no effective vaccines and drugs available. Here, one mouse-adapted CVA4 virus (YZ08) was cultivated by repeatedly cross-passaging between human RD cells and 11-day-old juvenile mice. Our results revealed the YZ08 virus was highly lethal to 13-day-old juvenile mice. Virus titer assay indicated that the YZ08 virus effectively replicated in juvenile mice. This model was successfully used to evaluate the therapeutic efficacy of herb medicine, anti-CVA4 rabbit sera, and antiviral drugs. Our results indicated that all tested reagents effectively provided protection for CVA4 virus infection by prolonging survival time, promoting survival, and preventing weight loss. Collectively, we successfully established a juvenile mouse model of CVA4 virus, which was a powerful tool for investigating the pathogenesis of CVA4 or evaluating the efficacy of antiviral reagents or vaccines.

## Highlights

• One mouse-adapted coxsackievirus A4 (CVA4) strain was cultivated
• A 11-day-old juvenile mouse CVA4 infection model has been successfully established
• This CVA4 mouse model was successfully used to evaluate the efficacy of antiviral reagents

## Graphical abstract

**Figure:**
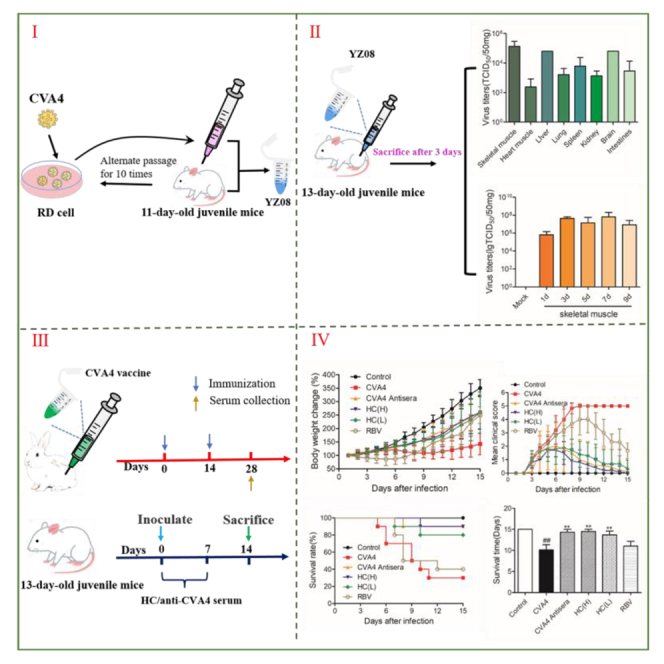

## Introduction

Coxsackievirus A4 (CVA4) is one of the important pathogens that cause hand, foot, and mouth disease (HFMD). It belongs to *picornavirus* family, *enterovirus* genus, and is a single-sense positive-strand RNA virus containing about 7,400 nucleotides.^1^ The first CVA4 virus strain was isolated in 1948 in the United States,^2^ and then it gradually became a global epidemic disease.^3^ During the past 10 years, it has been documented that CVA4 has become the dominant epidemiological pathogen of HFMD globally in children, which resulted in more severe and widespread epidemics.^4^ Due to its potential to cause pneumonia, myocarditis, meningitis, and even death in children, CVA4 has been widely considered by worldwide governments.^5^^,^^6^ Recently, several CVA4 outbreaks also occurred in China. For example, a clinical survey from Yunnan province indicated that CVA4 became one major causative enterovirus serotype causing HFMD in 2013.^7^ In 2019, CVA4-positive cases accounted for 7.8% of severe HFMD cases in Beijing.^8^ In 2021, a recombinant CVA4 virus was identified in a kindergarten in Shandong province.^9^ Due to the increasing severity and prevalence of CVA4 infection, CVA4 infection has become the major disease that seriously threaten the health of children worldwide.

Numerous studies have demonstrated that vaccines are the most effective approach for preventing enterovirus infections. For example, polio has been eliminated due to widespread vaccination.^10^ Furthermore, because of the widespread vaccination of EV71 and CVA16 inactivated vaccines in China, the incidence rate of EV71 and CVA16 declined year by year.^11^^,^^12^ However, there is currently no effective drugs for treating HFMD. Therefore, it is very urgent to develop innovative drugs for treating HFMD. An ideal animal model for mimicking clinical diseases is necessary for the development of antiviral drugs and vaccines. Recently, more and more attention has been paid to the development of animal models for HFMD. The animal models of enterovirus infection mainly include the non-primate model, tree shrew model, and mouse model.^13^^,^^14^^,^^15^ Non-primate models are often used to investigate the pathogenic mechanism of enterovirus due to their close relationship with humans. Recently, it has been reported that tree shrews, which present high homology with non-human primates, may be suitable for the CVA16 infection model.^14^ However, these models might not be suitable for antiviral drug efficacy evaluation because of the constraints of ethics, price, susceptibility, and scale.^16^ To our best knowledge, mice are currently the most widely used animal models for HFMD due to its availability and cost-effectiveness. It has been documented that some 1- to 7-day-old neonatal mice models have been infected with mouse-adapted or non-mouse-adapted EV71, CVA16, CVA10, CVA6, or CVA4 viruses for evaluating the effectiveness and safety of vaccine or antiviral drugs.^17^^,^^18^^,^^19^^,^^20^^,^^21^ For example, in the neonatal mouse CVA4 model, the infected mice became sick, paralyzed, and even died, and virus replication was confirmed *in vivo*. However, there are many defects such as small body weight, underdeveloped organs, and limited drug delivery capacity of 1- to 7-day-old suckling mice. These mice models may be unsuitable for evaluating the efficacy of antiviral drugs and vaccines. Therefore, it is very urgent to establish a mouse model for developing drugs and vaccines against CVA4 virus.

Here, a 13-day-old juvenile mice model has been successfully established with a mouse-adapted CVA4 strain, which resolves the limitations of previous models reliant on neonatal mice with underdeveloped organs and limited drug delivery capacity. The optimal age, infectious dose, and inoculation route were also determined. Thirteen-day-old juvenile mice intramuscularly inoculated with 100 TCID_50_ of CVA4 virus can produce consistent outcomes, including neurological symptoms, weight loss, and 100% mortality. The development of a reliable and scalable animal model is a valuable tool to enable more precise studies of CVA4 pathogenesis and the preclinical evaluation of antiviral candidates.

## Results

### Replication kinetics of CVA4 in RD cells

**Figure 1:**
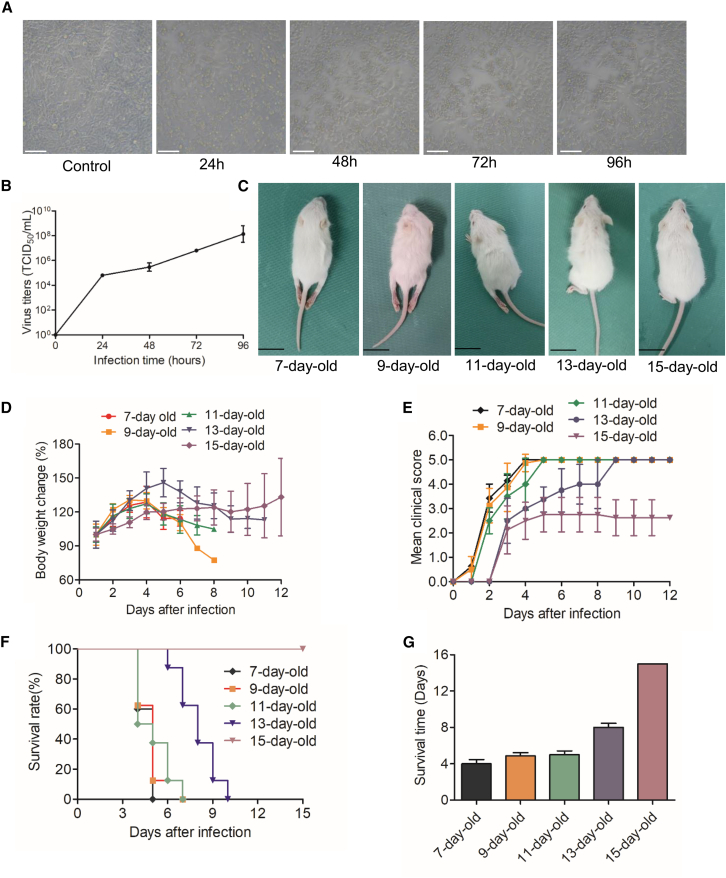
Pathogenicity of YZ08 virus in RD cells and mice (A) RD cells were infected with 0.01MOI of YZ08 CVA4 virus, and the representative images from one of three independent experiments were presented. Scale bars, 100μm. (B) RD cells were infected with 0.01MOI of YZ08 CVA4 virus. The virus titers in cell supernatants collected at the indicated time were determined by TCID_50_ assay. Data are the mean ± SD of three experiments. (C) 7-, 9-, 11-, 13-, and 15-day-old suckling mice were infected with 100 TCID_50_ of YZ08 CVA4 virus (*n* = 8), one representative image from each group photographed at the 5^th^ day post-infection were presented. (D)The body weight was recorded daily for 12 days. (E)The clinical symptoms of each mouse were recorded daily, and clinical scores were graded as follows: 0, healthy; 1, lethargy and inactivity; 2, ataxic; 3, weight loss; 4, limb paralysis; 5, dying or death. (F) The mortality was recorded daily, and the survival curve was created by Kaplan-Meier method using GraphPad Prism 5.00 (San Diego, California, USA), and the mean survival time of each group was recorded (G). Data are the mean ± SD of eight mice/group.

To further investigate the replication kinetics of CVA4 virus, the virus titers of cell supernatants inoculated with 100 TCID_50_ of CVA4 virus (0.1MOI) were determined. The virus titers were time-dependently increased (Figure 1). CVA4 virus resulted in 50% of cellular lesions and produced 10^4.8^ TCID_50_/mL at 24 h post-infection (hpi). After infection, RD cells exhibited cytopathic effects, including rounding, wrinkling, detachment, and necrosis (Figure 1A). At 96 hpi, the virus titers were gradually increased to 10^8.1^ TCID_50_/mL (Figure 1B).

### Pathogenicity in suckling mice of different age

To determine the reasonable age, 7- to 15-day-old mice were inoculated intramuscularly with 100 TCID_50_ units of CVA4 virus (Figure 1C). The body weight of 7- to 11-day-old mice began to decrease at 4^th^ day post-infection (dpi), whereas the weight loss of 13-day-old mice was delayed to 5 dpi (Figure 1D). All suckling mice exhibited severe clinical symptoms manifested as lethargy, reduced mobility, and limb paralysis at the death peak. However, compared with mice older than 11 days, 7- and 9-day-old mice exhibited more severe symptoms manifested as shorter illness onset time, shorter survival time, and higher mortality during the observation time (Figures 1E and 1F). All 7-, 9-, 11-, and 13-day-old CVA4-infected mice died during the observation period with different survival time (4, 4.87, 5, and 8 days, respectively) (Figure 1G). However, no death was observed in all 15-day-old CVA4-infected mice. Collectively, all above mentioned results indicated that YZ08 CVA4 mice-adapted strain exhibited highly pathogenicity in 7- to 13-day-old mice.

### Pathogenicity in 13-day-old juvenile mice

**Figure 2:**
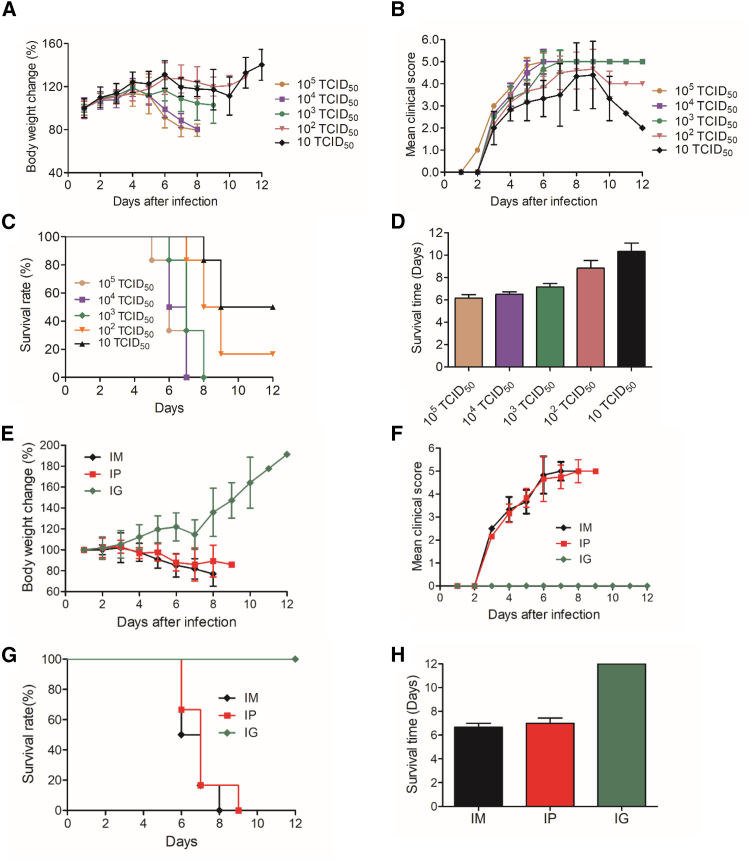
Determination of optional infection dose and route 13-day-old mice were infected with the indicated doses of YZ08 CVA4 virus (*n* = 10); their body weight (A) and clinical scores (B) of each group were recorded daily for 12 days, and the mortality (C) and survival time (D) were recorded for 15 days. Data are the mean ± SD of ten mice/group. (E) 13-day-old mice were inoculated by intramuscularly, intraperitoneally, and intragastrically with 100 TCID_50_ of YZ08 CVA4 virus (*n* = 10), and the clinical symptoms of each mouse were scored daily for 12 days (F). The mortality and mean survival time of each group were recorded daily for 15 days, and the survival curve (G) and survival time (H) were created by Kaplan-Meier method using GraphPad Prism 5.0 (San Diego, California, USA). Data are the mean ± SD of 10 mice/group.

To optimize the challenge dose, we further investigate the pathogenicity of YZ08 in 13-day-old juvenile mice. The severity of clinical symptoms was highly associated with the infectious dose (Figure 2A). Groups inoculated with 10^3^, 10^4^, and 10^5^ TCID_50_/mouse exhibited more severe clinical symptoms than 10^2^ and 10 TCID_50_-inoculated groups with shorter survival time, shorter initial death time, and higher mortality (Figure 2). The 50% lethal dose of YZ08 virus in 13-day-old ICR mice is 10 TCID_50_/0.1 mL. Thus far, 100 TCID_50_/0.1 mL was selected as the optimal challenge dose for establishing models.

### Pathogenicity of different infection routes

To determine the optimal inoculation route, 13-day-old mice were challenged by intramuscular (i.m.), intraperitoneal (i.p.), or intragastrical (i.g.) routes with 100 TCID_50_ of CVA4 virus, respectively (Figure 2E). In mice inoculated by i.m. or i.p. routes, clinical symptoms began to appear at 2 dpi, reached the highest clinical scores at 7 dpi, and their body weight began to decrease at 3 dpi, whereas the mice inoculated by i.g. route had no obvious clinical symptoms during the observation period (Figure 2F). All mice inoculated by i.m. and i.p. routes died at 8 dpi and 9 dpi, respectively, whereas no death in the i.g. group occurred, except for slight weight loss at 7 dpi (Figures 2G and 2H). The weight loss and clinical scores of the mice inoculated by the i.m. route were slightly higher than those of the mice inoculated by i.g routes, suggesting that i.m. and i.p. routes were the optional inoculation routes for establishing CVA4 infection models. Based on the comprehensive assessment of weight loss, disease symptoms, clinical scores, and survival time, the i.m. route was selected as the optimal infection route.

### Virulence in suckling mice of different ages

**Figure 3:**
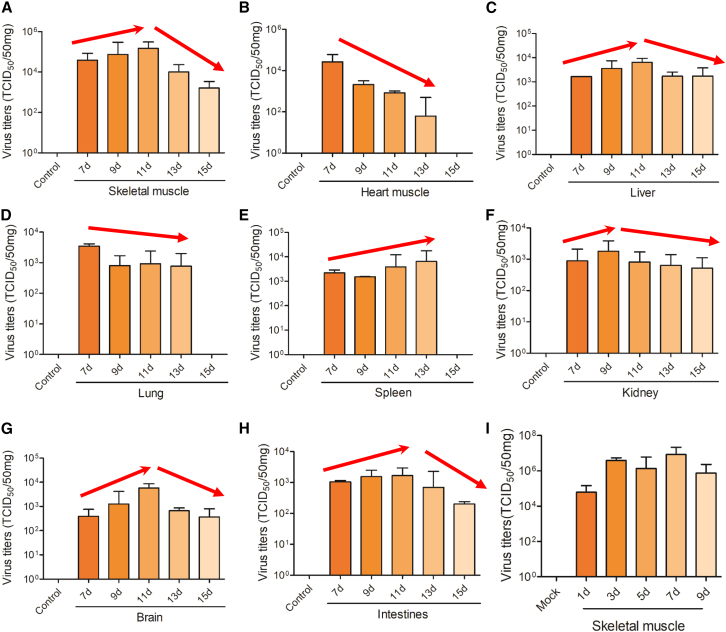
Virulence in 7- to 15-day-old mice 7- to 15-day-old mice were infected with 100 TCID_50_ of YZ08 CVA4 virus, respectively (*n* = 8). At the 3^rd^ of post-infection, six mice were randomly selected to euthanize, and the virus titers in skeletal muscle (A), heart (B), liver (C), lung (D), spleen (E), kidney (F), brain (G), and intestines (H) were determined using TCID_50_ assay. Data are the mean ± SD of eight mice/group. (I) Replication kinetics of YZ08 CVA4 virus in 13-old-day mice. 13-day-old mice were infected with 100 TCID_50_ of YZ08 CVA4 virus, (*n* = 8). The virus titers of skeletal muscle tissues were examined at 1, 3, 5, 7, and 9 dpi for virus titers assay. Data are the mean ± SD of eight mice/group.

To further assess the virulence of YZ08 CVA4 strain, the virus titers in various tissues including heart, lung, liver, kidney, brain, intestine, and skeletal muscle were examined (Figures 3A–3H). Our results revealed YZ08 virus can effectively replicate in all tissues of YZ08-infected mice, but the virus titers in skeletal muscle were the highest among all detected tissues. However, the phenotypic differences in virus replication levels in tissues of different ages of mice were observed. The virus replication levels in skeletal muscle, liver, brain, and intestines were age-dependently increased from 7- to 11-day-old mice but age-dependently decreased from 11- to 15-day-old mice. Moreover, the virus load in the heart and lung was age-dependently decreased from 7- to 13-day-old mice, whereas their replication levels in the spleen were age-dependently elevated from 7- to 13-day-old mice. In addition, the virus replication levels in the kidney were basically similar to those of tissues such as skeletal muscle, liver, and brain. However, in 15-day-old mice, we did not detect virus from heart, lung, and spleen at 3 dpi, which might be associated with the low pathogenicity of YZ08 CVA4 to 15-day-old mice. Collectively, all the abovementioned results indicated that CVA4 YZ08 virus can effectively replicate *in vivo* in 7- to 13-day-old mice.

To further confirm the muscle tropism of CVA4 YZ08 virus, we examined the replication kinetics of this virus in 13-day-old ICR mice. The viral titers in the skeletal muscles were gradually increased from 1 to 7 days after infection and reached the highest peak at 7 dpi (Figure 3I). But overall, the virus titers in muscle at 5, 7, and 9 dpi are maintaining high levels.

### CVA4 YZ08 caused excessive cytokine storm in mice

**Figure 4:**
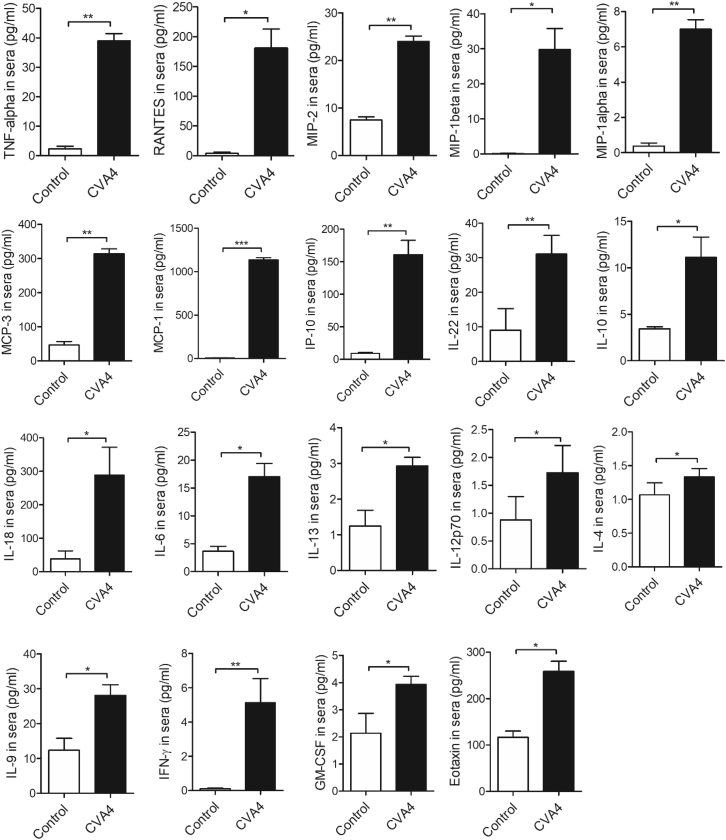
Serum cytokine assay 13-day-old mice were infected intramuscularly with 100 TCID_50_ of YZ08 CVA4 virus. The sera from all groups were collected at 3 dpi, serum cytokines and chemokines levels in mouse sera were measured using the Mouse Cytokine & Chemokine Panel 26Plex 96 tests. Data are the mean ± SD of 10 mice/group. Statistical analysis was performed by Student’s t test: ^∗^*p* < 0.05, ^∗∗^*p* < 0.01, ^∗∗∗^*p* < 0.001 control vs. CVA4-infected group.

To investigate whether YZ08 CVA4 causes excessive cytokine storms, we examined the serum inflammatory factors with multi-cytokine microarray skip. Compared with the control group, the levels of all examined 19 cytokines including macrophage inflammatory protein 2 (MIP-2), interleukin (IL)-22, IL-10, IL-18, tumor necrosis factor alpha (TNF-α), IL-6, IL-13, IL-9, IL-4, IL-12p70, interferon gamma (IFN-γ), MCP-1, MCP-3, MIP-1α, MIP-1β, RANTES, IFN-γ-inducible protein (IP-10), eotaxin, and granulocyte macrophage colony-stimulating factor (GM-CSF) in virus-infected group were significantly elevated, suggesting the excessive cytokine storm caused by CVA4 YZ08 in 13-day-old mice (Figure 4).

### Histopathological changes

**Figure 5:**
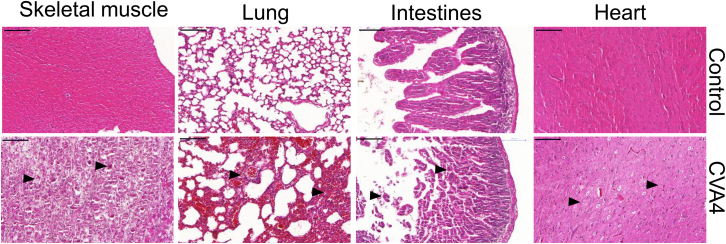
Histopathological examination 13-day-old mice were infected intramuscularly with 100 TCID_50_ of YZ08 CVA4 virus and their skeletal muscle, lung, intestines, and heart tissues were collected at 3 dpi for HE staining (*n* = 5). Scale bars, 100μm.

To further confirm the pathogenicity of CVA4 YZ08 virus, 13-day-old mice were inoculated intramuscularly with 100 TCID_50_/mouse. At 3 dpi, the mice were euthanized, and their heart, liver, spleen, lung, kidney, brain, intestines, and skeletal muscle were collected for pathological analysis (Figure 5). Pathological examination revealed severe muscle fiber necrosis and a large number of inflammatory cell infiltration in skeletal muscle. Moreover, severe lung lesions are characterized by broken alveolar walls, thickened alveolar septa, and alveolar wall infiltration of inflammatory cells. Small intestinal villi showed obvious congestion, ulceration of the small intestinal mucosa, and inflammatory cell infiltration. Many neuron cells in the brain were observed to be degenerated and necrotic, accompanied by inflammatory cell infiltration. However, no pathological changes in the tissues of the normal control group were observed, suggesting that CVA4 infection resulted in histopathological damage in 13-day-old juvenile mice.

### Evaluation of the protective effect of antiviral reagents against CVA4 virus

**Figure 6:**
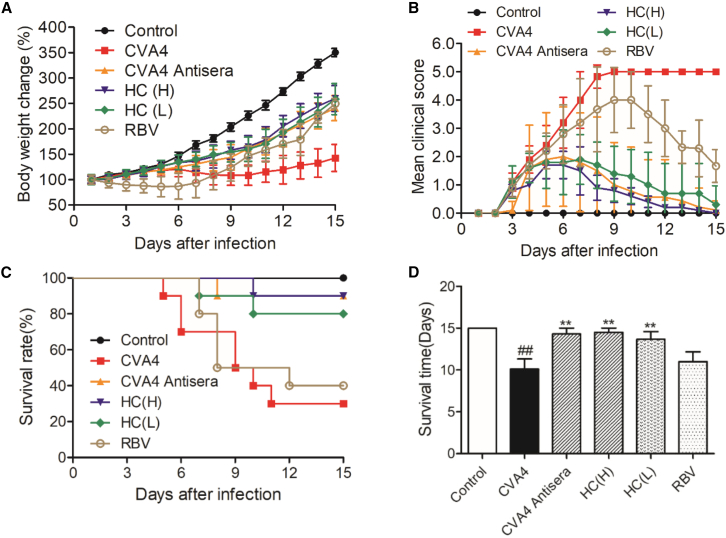
Evaluation of protective effect of antiviral reagents against CVA4 virus 13-day-old mice were infected with 100 TCID_50_ of YZ08 CVA4 virus. Two hours after infection, these mice were treated with water extract of HC, ribavirin, and anti-CVA4 rabbit sera for 6 days, respectively. Their body weight (A), clinical scores (B), survival rate (C), and survival time (D) of each group were recorded daily for 15 days. Data are the mean ± SD of 10 mice/group.

To investigate the reliability of this model, we evaluated the therapeutic efficacy of Chinese medicine *Houttuynia cordata* Thunb (HC), ribavirin, and anti-CVA4 serum against CVA4 virus (Figure 6). All CVA4-infected mice exhibited typical clinical symptoms such as lethargy, disinclination for activities, limb paralysis, or quadriplegia at 3 dpi and died within 13 dpi. Compared with the infected group, HC and anti-CVA4 serum treatment could significantly alleviate the clinical symptoms and dose-dependently provide protection on the lethal infection of CVA4 virus by prolonging the survival time, promoting mice survival, and improving the pathological injury. However, the positive drug ribavirin has only limited protective effect.

### Phylogenetic analysis

**Figure 7:**
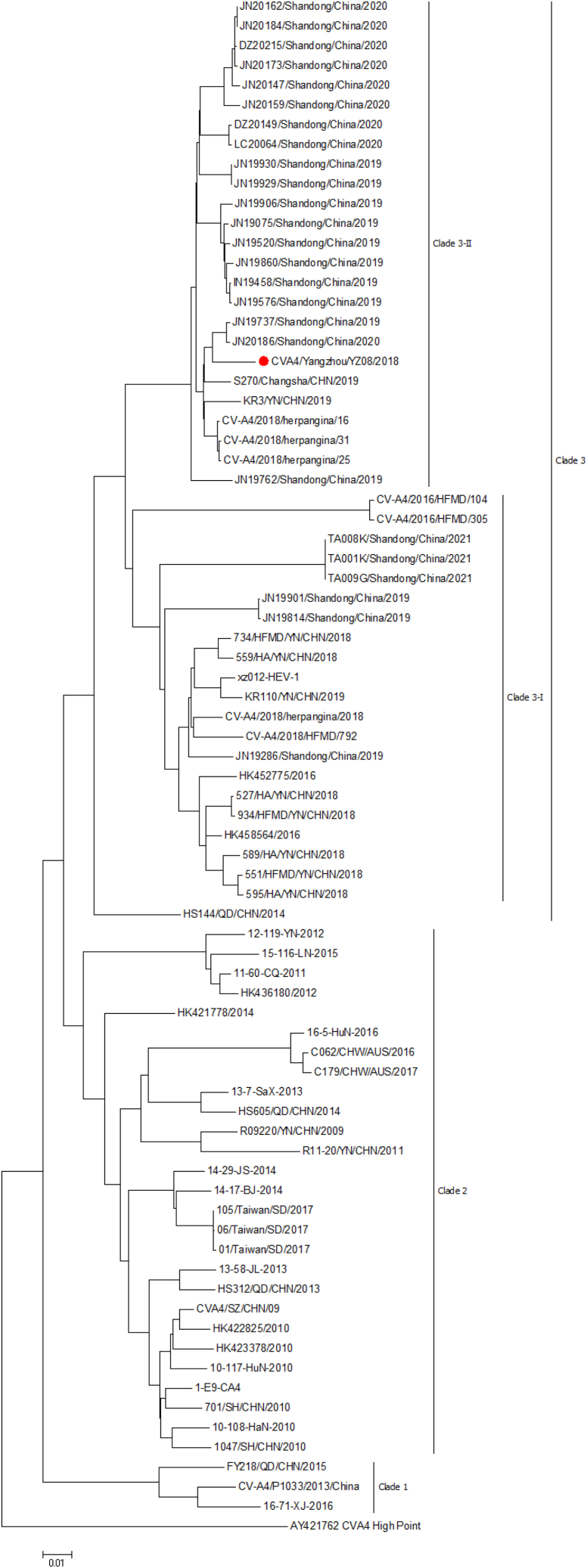
Phylogenetic tree of CVA4 viruses circulating in China during the past 5 years A maximum likelihood phylogenetic trees were constructed with MEGA7.0. The evolutionary distances were computed using the Maximum Composite Likelihood method.

Phylogenetic analysis based on the full genome indicated that all CVA4 strains isolated from China mainly clustered into three clades, including clade 1, clade 2, and clade 3 (Figure 7). Moreover, clade 3 was further divided into two branches, clade 3-I and clade 3-II. YZ08 virus was clustered into clade 3-II (genotype C) with these viruses isolated from Shandong, Hunan (Changsha), Yunnan, and Jiangsu provinces, which belong to the same genotype that resulted in herpangina outbreak epidemic in Jiangsu province in 2018 and 2019.

## Discussion

Due to the successful development and universal vaccination of EVA71 and CVA16 inactivated vaccines, HFMD caused by these two serotype viruses have been effectively controlled in China. However, CVA4, CVA6, and CVA10 have become the dominant pathogens in HFMD patients since 2009. According to the epidemiological surveillance of Yangzhou CDC, herpangina outbreaks caused by CVA4 occurred in Yangzhou, in spring 2019. Our results confirmed CVA4 was the most prevalent pathogen resulting in herpangina outbreak. Phylogenetic analysis indicated that the YZ08 virus was closely related to the most prominent CVA4 strains circulating in Beijing, Shandong, and Jiangsu during 2018–2020.^9^^,^^22^^,^^23^ Phylogenetic tree indicated that all CVA4 viruses circulating in China during 2010–2020 were clustered into three clades. YZ08 virus belonged to genotype C in clade 3-II with high nucleotide homology to these viruses causing herpangina outbreak in Jiangsu province during 2018–2019 (Figure 7).

Ideal animal models are important tools for investigating the pathogenesis of pathogens or the efficacy of innovative antiviral drugs and vaccine candidates. As the most cost-effective animal model, mice are widely considered as ideal animal models for investigating the pathogenesis of various enterovirus infections. There are some reports of CVA16, CVA10, CVA6, and CVA4 mice models.^24^^,^^25^^,^^26^ Although these reported HFMD models contributed to elucidating the pathogenesis of enteroviruses, they might not be an option for evaluating the efficacy of antiviral drugs and vaccines due to some limitations such as small body size, underdeveloped organs, and limited drug delivery capacity. Here, we have established a 13-day-old juvenile mice model with a mouse-adapted CVA4 virus. In this model, all typical neurological symptoms such as hind limbs, forelimbs, three limbs, and quadriplegia paralysis have been observed. Moreover, severe pathological damage and excessive cytokine storm in infected mice were observed in this model. Furthermore, this model has been successfully used to evaluate the efficacy of *Houttuynia cordata* (HC), anti-CVA4 serum, and ribavirin, which demonstrated that HC and anti-CVA4 serum improved survival, reduced weight loss, and mitigated pathological injury, whereas ribavirin showed limited efficacy. The study holds significant relevance for researchers in infectious disease and virology, particularly those focused on enteroviruses and hand, foot, and mouth disease (HFMD). The development of a reliable and scalable animal model is a valuable contribution to this field, enabling more precise studies of CVA4 pathogenesis and the preclinical evaluation of antiviral candidates. The study has broader implications for translational research, particularly for pharmacologists, immunologists, and drug developers.

Ribavirin as a broad-spectrum antiviral nucleoside analog has been widely used in clinical practice for the treatment of viral infectious diseases. Moreover, it has been demonstrated that ribavirin could provide protective effect in the early stages of CVA6 infection by increased survival in 5-day-old neonatal mice.^27^ However, our results indicated the protective effect of ribavirin against CVA4 infection was very limited. Furthermore, the weight loss in ribavirin-treated group was greater than that of CVA4-infected group, which might be associated with the side effect of ribavirin. Vaccination is considered as the most effective approach for controlling HFMD. The CVA4 antisera prepared by the inactivated YZ08 virus vaccine can provide 100% protection for the lethal infection of CVA4 and prevented the cell pathogenic effect of CVA4 virus in RD cells. *Houttuynia cordata* Thunb (HC), which is a Chinese herbal medicine with homology of medicine and food known as the “broad-spectrum antibiotic of traditional Chinese medicine” has been widely used to treat infectious diseases in China.^28^ It has been reported that the water extract and polysaccharide of HC has antiviral activity against coxsackievirus B3 (CV-B3), coxsackievirus B5 (CV-B5), and enterovirus 71 (EV71) *in vitro*.^29^ However, whether HC has an antiviral effect on CVA4 remains unclear. Here, our results indicated that HC exhibited promising anti-CVA4 activities. The confirmed efficacy of HC highlights the potential of natural products and Chinese traditional medicine in addressing viral diseases, aligning with global efforts to diversify therapeutic pipelines.

In summary, a 13-day-old juvenile mouse for CVA4 infection has been successfully established to address limitations of previous models reliant on neonatal mice with underdeveloped organs and limited drug delivery capacity, which will enable more precise studies of CVA4 pathogenesis and the preclinical evaluation of antiviral candidates.

### Limitations of the study

Although this CVA4-infected juvenile mice model has been successfully established, several questions remain unanswered: (1) the potential pathogenesis of this CVA4 (YZ08) to juvenile mice remains to be elucidated in the future; (2) what is the underlying mechanism by which CVA4 (YZ08) loses the ability to successfully infect mice via the oral route; (3) whether this CVA4 infection mice can mimic human-like symptoms in the oral cavity remains to be investigated; (4) whether this CVA4 can infect other mouse strains needs to be confirmed; (5) the exact underlying mechanisms of HC against CVA4 infection remains unclear; (6) whether the model’s applicability to human diseases remains to be investigated in the future; (7) the differential susceptibility to CVA4 virus (YZ08) between sexes has not yet been investigated.

## Resource availability

### Lead contact

Further information and requests for resources and reagents are available from the lead contact, Pinghu Zhang (zhangpinghu@163.com).

### Materials availability

The viruses and materials used in this study can be obtained from the lead contact after the permission of original distributors.

### Data and code availability

• All data reported in this study will be shared by the lead contact upon request.
• This study does not report original code.
• Any additional information required to reanalyze the data reported in this study is available from the lead contact upon request.

## Acknowledgments

This work was supported by the Jiangsu Province Traditional Chinese Medicine Technology Development Plan Project (MS2022155). We greatly appreciate all supports including research conditions and technical supports kindly provided by Drs. Xiaoquan Wang, Xuhua Mao, and Weiqing Shi.

## Author contributions

Q.S., X.C., Z.Z., H.W., J.X., L.Z., and A.M. investigation; Q.S., manuscript drafting; X.C., Y.W., and X. C., critical resources; Y.W., X.C., and P.Z. conceptualization and supervision. P.Z. manuscript writing and editing.

## Declaration of interests

All authors declare no competing interests.

## STAR★Methods

### Key resources table

**Table:** 

REAGENT or RESOURCE	SOURCE	IDENTIFIER
**Virus strains**		

CVA4/YZ08/Yangzhou/2019 (YZ08)	Yangzhou University	Pinghu Zhang

**Chemicals, peptides, and recombinant proteins**		

MEM	Key GEN	Cat# KGM12800N-500
Fetal bovine serum	Capricorn Scientific	Cat# FBS-ES-22A
0.25% trypsin	New Cell	Cat# C100C1
Crystal violet	Solarbio	Cat# C8470
RNA isolater	Vazyme	Cat# R401-01
HiScript II 1st Strand cDNA Synthesis Kit	Vazyme	Cat# R211-01
2×Phanta Max Master Mix	Vazyme	Cat# P515-01
DNA Marker	Vazyme	Cat# MD101-02
Penicillin-streptomycin-amphotericin B solution	Beyotime	Cat# C0224
TAE (50×)	Beyotime	Cat# ST717
Agarose gel PCR purification and recovery kits	Promega	Cat# A9281
Super Red nucleic acid dye	Biosharp	Cat# BS354B
Primers	Gene script	N/A

**Experimental models: Cell lines**		

RD	CCTCC	GDC0295

**Oligonucleotides**		

PCR primers for CVA41-636 (see Table S1)	Nanjing Genscript Bio, Inc.	N/A
PCR primers for CVA4524-1135 (see Table S1)	Nanjing Genscript Bio, Inc.	N/A
PCR primers for CVA4 987-1717 (see Table S1)	Nanjing Genscript Bio, Inc.	N/A
PCR primers for CVA4 1635-2187 (see Table S1)	Nanjing Genscript Bio, Inc.	N/A
PCR primers for C-VA4 2086-2840 (see Table S1)	Nanjing Genscript Bio, Inc.	N/A
PCR primers for CVA4 2689-3324 (see Table S1)	Nanjing Genscript Bio, Inc.	N/A
PCR primers for CVA4 3233-3882 (see Table S1)	Nanjing Genscript Bio, Inc.	N/A
PCR primers for CVA4 3714-4467 (see Table S1)	Nanjing Genscript Bio, Inc.	N/A
PCR primers for CVA4 4312-5104 (see Table S1)	Nanjing Genscript Bio, Inc.	N/A
PCR primers for CVA4 4997-5974 (see Table S1)	Nanjing Genscript Bio, Inc.	N/A
PCR primers for CVA4 5822-6503 (see Table S1)	Nanjing Genscript Bio, Inc.	N/A
PCR primers for CVA4 6384-7287 (see Table S1)	Nanjing Genscript Bio, Inc.	N/A
PCR primers for CVA4 7137-7420 (see Table S1)	Nanjing Genscript Bio, Inc.	N/A

**Software and algorithms**		

GraphPad Prism	GraphPad Prism version 5.00	https://www.graphpad.com/features
LaserGene	DNASTAR	https://www.dnastar.com/software/
MEGA	MEGA7.0	https://www.megasoftware.net/

**Deposited data**		

CVA4/YZ08/Yangzhou/2019 (YZ08)	GenBank: PV002720	GenBank

### Experimental model and study participant details

#### Cell culture and viruses

Rhabdomyosarcoma (RD) cell line (mycoplasma negative) was purchased from China Center for Type Culture Collection of Wuhan University (CCTCC: GDC0295) and was cultured with minimum essential medium (MEM) supplemented with 10% fetal bovine serum. This CVA4 clinical strain was isolated with RD cells from the Affiliated Hospital of Yangzhou University. The 50% tissue culture infection dose (TCID_50_/50μl) were determined with the Reed and Muench method.^30^ CVA4/YZ08/Yangzhou/2019 (YZ08) virus was domesticated by cross-passage between 11-day-old suckling mice and RD cells. YZ08 virus is unable to replication in Vero cells *in vitro*. To prepare the viral stocks, one dish (10 cm) of RD cells were infected with 100 TCID_50_ of YZ08 virus and cultured for 72 h. The supernatant was collected and centrifuged with 12000 rpm for 10 min at 4°C, and then aliquoted and stored at -80°C.

### Method details

#### Sequencing and phylogenetic analysis

Viral RNA was extracted as follows. Briefly, 200 μl of virus supernatants were added into 1ml of Trizol to lysis the virus, and then added 200 μl of chloroform. After completely mixing and shaking, the samples were centrifuged at 12000 rpm for 10 min at 4°C. 550 μl of the upper water phase was collected, and then mixed with the same volume of isopropanol, and centrifuged at 12000 rpm for 10 min at 4°C again to remove the supernatant. The viral RNA was washed with 75% ethanol for once, and then 40 μL of DEPC water was added to dissolve the viral RNA. The extracted RNA was stored at -80°C. The extracted RNA was reversed into cDNA with HiScript II 1st Strand reverse transcription kit according to the manufacturer’s manual (Vazyme, Nanjing, China). Briefly, 7μl viral RNA were mixed with 1 μl Oligo T primers and incubated at 70°C for 10 min, and then mixed with 10 μl of reverse buffer and 2 μl of reverse enzyme mix. Subsequently, the viral RNA was reversed as following protocols: 25°C for 10 min, 55°C for 60 min, and then 85°C for 2 min. Using the above reversed cDNA as a template, 13 pairs of designed primers were used for PCR, respectively, and the viral genome cDNA fragments were amplified in segments as following reaction conditions: pre-denaturing at 95min°C for 5 min; denaturing at 95°C for 1 min, annealing at 60°C for 1 min, and then extending at 72°C for 2 min for 35 cycles; 72°C extending for 10min. The PCR products were separated by 1% agarose gel electrophoresis, and the target fragments were recovered by agarose gel PCR purification and recovery kits (Promega, USA) after gel cutting, and then the PCR product were sent to Sangon Biotech (Shanghai, China) for sequencing. The complete genome sequences of CVA4 reported in 2018-2022 were downloaded from NCBI, and the sequences were analyzed by using DNASTAR software. The specific primers were designed using Primer 5.0 primer according to the highly conserved nucleic acid regions of CVA4 viruses. The primers are shown in Table S1. The sequence was assembled into the full-length sequence by DNASTAR. Full genome sequence of this virus was submitted to the GenBank of National Center for Biotechnology Information (accession number PV002720). A maximum likelihood phylogenetic tree based on full genome of CVA4 viruses was constructed with MEGA7.0.

#### Replication kinetics

RD cells were seeded into a 6-well plate with 1.0×10^6^ cells/well. After washing twice with phosphate-buffered saline (PBS), the plates were infected with 100 TCID_50_ of YZ08 CVA4 virus for 1h at 37°C, and then were washed with PBS twice. The supernatants were replaced with maintenance MEM medium and incubated at 37°C, 5% CO_2_ for 96h. Cell supernatants were collected at the indicated time, and virus titers (TCID_50_/mL) were determined by TCID_50_ assay.^17^

#### Ethics statement

All ICR mice were purchased from Comparative Medical Center for Yangzhou University and housed under specific-pathogen-free conditions in ventilated cages (IVC, Tecniplast, Milan, Italy) of Yangzhou University on a 12 h light/dark cycle and maintained at 22 ± 2°C with *ad libitum* access to food and water. All animal experiments were strictly conducted according to the guidelines of Jiangsu laboratory animal welfare and all experimental protocols were approved by the Ethics Committee of Yangzhou University (permission no: 202408022). To alleviate the pain, all mice were lightly anesthetized with isoflurane.

#### Animal experiments

To investigate the reasonable age, 7-, 9-, 11-, 13- and 15-day-old ICR mice from eight litters (n=8 per group) were lightly anesthetized with isoflurane and inoculated intramuscularly with 100 TCID_50_ units of CVA4/mouse in 50 μL of PBS, respectively. To optimize the appropriate infectious dose, 13-day-old ICR mice from five litters were randomly assigned to five groups and inoculated intramuscularly with 10^1^, 10^2^, 10^3^, 10^4^, 10^5^ TCID_50_ units of virus/per mouse, respectively (n=10/group). To determine the appropriate inoculation routes, 13-day-old ICR mice from three litters were randomly assigned to three groups and were inoculated intramuscularly (i.m.), intraperitoneally (i.p.) and intragastrically (i.g.) with 100 TCID_50_ units of CVA4 virus suspension/mouse, respectively. To investigate the virulence of YZ08 virus in different age of mice, 7-, 9-, 11-, 13- and 15-day-old ICR mice from 5 litters were infected intramuscularly with 100 TCID_50_ units of CVA4 virus suspension. After infection, the mice body weight and mortality (death or loss of >25% of their beginning body weight) were monitored for 15 days. The 50% mouse lethal dose (LD_50_) was recorded as previously described.^24^ To investigate the reliability of this model, six litters of 13-old-day mice (10 mice/litter) were randomly assigned to 6 groups and infected with intramuscularly (i.m.) with 100 TCID_50_ of CVA4 virus /mouse. After infection for 2 h, six groups of mice were treated with Chinese medicine *Houttuynia cordata* Thunb. (HC), ribavirin, and anti-CVA4 serum, respectively. All animals were recorded daily for clinical signs of ruffled fur, weight loss, and limb paralysis. Clinical scores were graded as follows: 0, healthy; 1, lethargy and inactivity; 2, ataxic; 3, weight loss; 4, limb paralysis; 5, dying or death. Data are represented as mean +/- SEM.

#### Serum cytokine and pathology assay

13-day-old ICR mice were infected intramuscularly with 100 TCID_50_ units of CVA4 virus (n=10/group). At 5 days post-infection, 6 mice of each group were randomly euthanized and blood samples were collected for cytokines assay including IL-22, IL-10, IL-18, IL-6, IL-13, IL-9, IL-4, IL-12p70, IP-10, eosinophil chemotactic factor (Eotaxin), granulocyte-macrophage colony-stimulating factor (GM-CSF), interferon-gamma (IFN-γ), monocyte chemotactic protein-1 (MCP-1), monocyte chemotactic protein-3 (MCP-3), activated regulatory normal T cell expression and secretion factor (RANTES), macrophage inflammatory protein-1alpha (MIP-1α), macrophage inflammatory protein-1β (MIP-1β), macrophage inflammatory protein-2 (MIP-2), and TNF-α with Multi-Cytokine Microarray Kit according to the manufacturer's protocol as previously described.^31^ Data are represented as mean +/- SEM.Tissue samples from skeletal muscles, lungs, small intestines and brains were fixed with 10% neutral-buffered formalin and then routinely processed and embedded in paraffin, sectioned, and stained with hematoxylin and eosin for histopathological analysis.

#### Preparation of water extract of *Houttuynia cordata* Thunb

Dried plant material (50 g) was immersed in 20 times water for 1 h, boiled for 1 h, and filtered with gauze to remove drug residues and collect the filtrate. And then 2 L of water was added to the drug residues for boiling 1 h again and filtered to collect the filtrate again. All filtrate was mixed completely and concentrated to get 1 g/ml of *Houttuynia cordata* Thunb water extract.

#### Anti-CVA4 virus rabbit sera

The inactivated CAV4 vaccine was produced by inactivation of ZY08 virus. Briefly, the YZ08 virus were inoculated into RD cells for viral proliferation. The virus stock (10^8.8^TCID_50_/ml) was inactivated by adding 3% formaldehyde for 24 h, and then was emulsified with Freund's complete adjuvant in equal amounts (1:1) to prepare inactivated CVA4 virus vaccine. 0.5 mL of inactivated CVA4 vaccine was inoculated into the left and right legs of rabbits, respectively. Two weeks after inoculation, the rabbits were boosted with the same dose of inactivated vaccine once. Four weeks after inoculation, all immunized rabbits were sacrificed by bleeding and the sera were collected by centrifuging 5000 rpm for 10 min at 4°C. All sera were stored at -80°C for later use. The titer of anti-CVA4 serum was 1: 100 by neutralization test.

#### Hematoxylin and eosin (H&E) staining

The heart, lung, skeletal muscle, and intestine tissues from the CVA4-infected mice and the control were fixed with 10% formalin, then dehydrated through an ethanol gradient, and embedded in paraffin, followed by sectioning into 5-μm sections. Then, these sections were sequentially dewaxed, rehydrated, and stained with hematoxylin for 5 min, and then stained with eosin. After transparency, all sections were sealed and were observed in random visual fields under an under inverted microscope.

### Quantification and statistical analysis

All results were expressed as the mean ± standard deviation (SD) or median with range (non-normal distribution) of triplicate determinations. Statistical differences were performed using GraphPad Prism version 5.00 for Windows (GraphPad Software, San Diego, California USA, www.Graphpad.com) to analyze the difference in survival rate, weight, mean clinical scores between different groups. The survival curves were plotted using the Kaplan-Meier method and the differences of survival rates were assessed with the Mantel-Cox Log-rank test. The variance analysis was conducted by two-tailed Student’s t-test or Mann-Whitney test (non-normal distribution) on performing the comparison of two groups. The ^∗^*P*<0.05, ^∗∗^*P*<0.01, or ^∗∗∗^*P*<0.001 were considered as significant.

## References

[1] Wang M., Li J., Yao M.X., Zhang Y.W., Hu T., Carr M.J., Duchêne S., Zhang X.C., Zhang Z.J., Zhou H. (2019). Genome Analysis of Coxsackievirus A4 Isolates From Hand, Foot, and Mouth Disease Cases in Shandong, China. Front. Microbiol..

[2] Oberste M.S., Peñaranda S., Maher K., Pallansch M.A. (2004). Complete genome sequences of all members of the species Human enterovirus A. J. Gen. Virol..

[3] Zhu P., Ji W., Li D., Li Z., Chen Y., Dai B., Han S., Chen S., Jin Y., Duan G. (2023). Current status of hand-foot-and-mouth disease. J. Biomed. Sci..

[4] Ji T., Guo Y., Lv L., Wang J., Shi Y., Yu Q., Zhang F., Tong W., Ma J., Zeng H. (2019). Emerging recombination of the C2 sub-genotype of HFMD-associated CV-A4 is persistently and extensively circulating in China. Sci. Rep..

[5] Nassef C., Ziemer C., Morrell D.S. (2015). Hand-foot-and-mouth disease: a new look at a classic viral rash. Curr. Opin. Pediatr..

[6] Yamamoto H., Isogai J. (2023). Transient constrictive pericarditis following coxsackievirus A4 infection as a rare cause of acute mediastinitis: A case report. Heliyon.

[7] Du Z., Zhao Y., Luo Y., Du L., Gan Q., Zhang H., Li J., Yang Z., Ma S. (2019). Ongoing change of severe hand, foot, and mouth disease pathogens in Yunnan, China, 2012 to 2016. J. Med. Virol..

[8] Xie M.Z., Chen L.Y., Yang Y.N., Cui Y., Zhang S.H., Zhao T.S., Zhang W.X., Du J., Cui F.Q., Lu Q.B. (2022). Molecular Epidemiology of Herpangina Children in Tongzhou District, Beijing, China, During 2019-2020. Front. Med..

[9] Li J., Ni N., Cui Y., Zong S., Yao X., Hu T., Cao M., Zhang Y., Hou P., Carr M.J. (2022). An outbreak of a novel recombinant Coxsackievirus A4 in a kindergarten, Shandong province, China, 2021. Emerg. Microbes Infect..

[10] Duintjer Tebbens R.J., Thompson K.M. (2017). Poliovirus vaccination during the endgame: insights from integrated modeling. Expert Rev. Vaccines.

[11] Liang Z., Wang J. (2014). EV71 vaccine, an invaluable gift for children. Clin. Transl. Immunology.

[12] Liao Y., Jiang Q., Huo X., Yu L., Yang J., Zhao H., Li D., Xu X., Jiang G., Zhang C. (2023). Preclinical safety evaluation of a bivalent inactivated EV71-CA16 vaccine in mice immunized intradermally. Hum. Vaccin. Immunother..

[13] Duan S., Yang F., Li Y., Zhao Y., Shi L., Qin M., Liu Q., Jin W., Wang J., Chen L. (2022). Pathogenic analysis of coxsackievirus A10 in rhesus macaques. Virol. Sin..

[14] Li J.P., Liao Y., Zhang Y., Wang J.J., Wang L.C., Feng K., Li Q.H., Liu L.D. (2014). Experimental infection of tree shrews (Tupaia belangeri) with Coxsackie virus A16. Zool. Res..

[15] Qian S.S., Wei Z.N., Jin W.P., Wu J., Zhou Y.P., Meng S.L., Guo J., Wang Z.J., Shen S. (2021). Efficacy of a coxsackievirus A6 vaccine candidate in an actively immunized mouse model. Emerg. Microbes Infect..

[16] Yi E.J., Shin Y.J., Kim J.H., Kim T.G., Chang S.Y. (2017). Enterovirus 71 infection and vaccines. Clin. Exp. Vaccine Res..

[17] Chen Y., Li H., Yang J., Zheng H., Guo L., Li W., Yang Z., Song J., Liu L. (2021). A hSCARB2-transgenic mouse model for Coxsackievirus A16 pathogenesis. Virol. J..

[18] Gu J., Wu J., Cao Y., Zou X., Jia X., Yin Y., Shen L., Fang D., Mao L. (2020). A Mouse Model for Infection with Enterovirus A71 in Small Extracellular Vesicles. mSphere.

[19] Jin Y., Sun T., Zhou G., Li D., Chen S., Zhang W., Li X., Zhang R., Yang H., Duan G. (2021). Pathogenesis Study of Enterovirus 71 Using a Novel Human SCARB2 Knock-In Mouse Model. mSphere.

[20] Sun Q., Li J., Wang R., Sun T., Zong Y., Wang C., Liu Y., Li X., Song Y., Zhang Y. (2023). Coxsackievirus A6 Infection Causes Neurogenic Pathogenesis in a Neonatal Murine Model. Viruses.

[21] Zhou Y., Zhang C., Liu Q., Gong S., Geng L., Huang Z. (2018). A virus-like particle vaccine protects mice against coxsackievirus A10 lethal infection. Antiviral Res..

[22] Zhao T.S., Du J., Li H.J., Cui Y., Liu Y., Yang Y., Cui F., Lu Q.B. (2020). Molecular epidemiology and clinical characteristics of herpangina children in Beijing, China: a surveillance study. PeerJ.

[23] Guo W.P., Chen G.Q., Xie G.C., Du L.Y., Tang Q. (2020). Mosaic genome of Human Coxsackievirus A4 associated with herpangina and HFMD in Yancheng, China, 2016 and 2018. Int. J. Infect. Dis..

[24] Yang L., Mao Q., Li S., Gao F., Zhao H., Liu Y., Wan J., Ye X., Xia N., Cheng T., Liang Z. (2016). A neonatal mouse model for the evaluation of antibodies and vaccines against coxsackievirus A6. Antiviral Res..

[25] Li S., Zhao H., Yang L., Hou W., Xu L., Wu Y., Wang W., Chen C., Wan J., Ye X. (2017). A neonatal mouse model of coxsackievirus A10 infection for anti-viral evaluation. Antiviral Res..

[26] Zhang Z., Zhang X., Carr M.J., Zhou H., Li J., Liu S., Liu T., Xing W., Shi W. (2019). A neonatal murine model of coxsackievirus A4 infection for evaluation of vaccines and antiviral drugs. Emerg. Microbes Infect..

[27] Zhang Z., Dong Z., Li J., Carr M.J., Zhuang D., Wang J., Zhang Y., Ding S., Tong Y., Li D., Shi W. (2017). Protective efficacies of formaldehyde-inactivated whole-virus vaccine and antivirals in a murine model of coxsackievirus A10 infection. J. Virol..

[28] Wei P., Luo Q., Hou Y., Zhao F., Li F., Meng Q. (2024). Houttuynia Cordata Thunb.: A comprehensive review of traditional applications, phytochemistry, pharmacology and safety. Phytomedicine.

[29] Fan L., Hou L., Liu M., Tian J. (2019). Study on antiviral activity of different solvent extracts of Houttuynia Cordata. Chin J Mod Appl Pharm.

[30] Zhang Y., Wang R., Shi W., Zheng Z., Wang X., Li C., Zhang S., Zhang P. (2021). Antiviral effect of fufang yinhua jiedu (FFYH) granules against influenza A virus through regulating the inflammatory responses by TLR7/MyD88 signaling pathway. J. Ethnopharmacol..

[31] Wang W., Zheng Z., Qi X., Wei H., Mao X., Su Q., Chen X., Feng Y., Qiao G., Ma T. (2024). Clinical efficacy of Fufang Yinhua Jiedu (FFYH) granules in mild COVID-19 and its anti-SARS-CoV-2 mechanism by blocking autophagy through inhibiting the AKT/mTOR signaling pathway. Front. Pharmacol..

