# Supplementary material for: A lethal juvenile mouse model for the evaluation of antiviral reagents against coxsackievirus A4
Source: iScience. 2025 Jun 30;28(8):113022. doi: 10.1016/j.isci.2025.113022 (PMC12304755; doi:10.1016/j.isci.2025.113022)
Supplement: Document S1. Table S1 [file mmc1.pdf]

iScience, Volume *28*

Supplemental information

A lethal juvenile mouse model for the evaluation of antiviral reagents against coxsackievirus A4

Qin Su, Xiao Chen, Zhihui Zheng, Hailin Wei, Jiaxue Zhou, Libing Zhang, Ali Muhammad, Yin Wang, Xiang Chen, and Pinghu Zhang

Table. S1

Primer name	Primer sequence (5'- 3')	locus (nt)
CVA4 1F	GGTTGCACCCACTCACAGG	1-19
CVA4 1R	TACGATTGCTCTGTTAGACACCG	658-636
CVA4 2F	AACTCTGCAGCGGAACCGA	524-540
CVA4 2R	GTCCAAGACTTAGTGGATAATGTGTA	1160-1135
CVA4 3F	CCAATTAACTGTAGGAAACTCTAC	987-1010
CVA4 3R	GGTTTCATCTCAACAGGGAT	1736-1717
CVA4 4F	CACCACTGAGATCCCAATTACCA	1635-1657
CVA4 4R	ACAAGAGTGACTGACGACTGCAAT	2210-2187
CVA4 5F	GCAACAGGTAAGATGCTTATAG	2086-2107
CVA4 5R	CTAGATTGGTAACGAAGGTGA	2862-2840
CVA4 6F	TCTTCTCCAGGTCAGGATTAGT	2689-2710
CVA4 6R	GTTGTTCAGCGTTGTAGTCAGT	3345-3324
CVA47F	CCTTGAGATCACAACCATACATC	3233-3254
CVA4 7R	AAGGTAACTCTTGAGAGCTTCTACTT	3907-3882
CVA4 8F	TTGTTGGCATAGTGTCCACTG	3714-3735
CVA4 8R	TGATGATGAGACATACAGGTTCAA	4490-4467
CVA4 9F	CTCACAGGAAGACCTTGAAGCA	4312-4333
CVA4 9R	CTGATCCTGATTGGTCTGAACTTG	5127-5104
CVA4 10F	TCTAAGGTTAGGTATAGCGTGGACA	4997-5021
CVA4 10R	GGATCTCTCCCTGTTCACTAGCA	5996-5974
CVA4 11F	CCAACTCACCGTACTATGATGTAC	5822-5848
CVA4 11R	CTGGCTTCAATCAGTCGAGA	6522-6503
CVA4 12F	CTACCACTAGAGATGTGAGCAAGAT	6384-6408
CVA4 12R	GGAACAGACCTAATTGTGCTCAC	7310-7287
CVA4 13F	CAATTTCCATTCTTGATCCAC	7137-7157
CVA4 13R	TTGCTATTCTGGTTATAACAAATT	7420-7397
